# *Prevotella Copri* and Microbiota in Rheumatoid Arthritis: Fully Convincing Evidence?

**DOI:** 10.3390/jcm8111837

**Published:** 2019-11-01

**Authors:** Lorenzo Drago

**Affiliations:** Laboratory of Clinical Microbiology & Microbiome Unit, Department of Biomedical Sciences for Health, “Invernizzi” Pediatric Clinical Research Center, University of Milan, 20133 Milan, Italy; lorenzo.drago@unimi.it

## Abstract

Gut microbiota regulates the host’s immune system. Microorganisms and their compounds can co-exist peacefully with the immune system and coordinate its function and regulation. Some microbial clusters may be harmful and others helpful in the respective negative or positive balance of the immune network. These insights have revealed important mechanisms for understanding and treating autoimmune and inflammatory diseases. This Editorial aims to clarify the role of specific genus of gut microbiota, such as *Prevotella*, in influencing the pathogenesis of Rheumatoid Arthritis (RA).

Convincing studies propose microbiota as metabolites pathway regulators capable of exerting local and multi-level systemic long-lasting effects. One of these effects is very intriguing and includes the interaction with the immune system. Gut microbiota alone, indeed, is able to play an important role in the differentiation of immune cells, and this effect involves the entire body if we consider that 70% of the immune system is located in the gastrointestinal tract. Gut microbiota modulates, directly or indirectly (via metabolites production): (a) the innate and the adaptive system by the immunoregulation of T-cells, (b) the cytokines network, and (c) the functionality of dentritic cells and macrophages [1,2,3]. Thus, it is fully convincing that alterations in the gut microbiota can raise in inflammatory and autoimmune disorders [4,5].

Microbial colonization occurs at beginning of life, probably even during fetal condition, and continues to change and to shape in a dynamic way until its stabilization at around 3 years of age [6]. Looking at various properties of gut microbes, the microbiota can bind nod-like receptors (NDRs) and toll-like receptors (TLRs) to activate the immune system, and to produce some metabolites, such as the short-chain fatty acids (SCFAs), that can directly interact with the host [7,8]. The appropriate balance of microbiota in terms of variability and richness is able to maintain an adequate immune response and a tolerant state within the gastrointestinal tract. Recently, the correlation between the microbiota and the immune system in health and diseases has been well established [9]. This connection also involves Rheumatoid Arthritis (RA). The first publication on the link between microbiota and the pathogenesis of arthritis in an experimental animal model was published in the late 1970s [10]. The study observed that germ-free rats developed more severe arthritis in all the cases compared to the conventional rats harboring a normal gut microbiota. The precise role of the gut microbiota-dysbiosis in the causation of RA has been further studied in mice expressing the *DRB1* susceptible RA gene [11]. 

Recently, human studies have demonstrated that low diversity and dysbiosis of gut microbiota can have a key role in the evolution of RA [12,13]. In particular, these original studies mentioned the role of *Prevotella copri* in the RA pathogenesis. More recently, a study [14] has clearly shown that the microbial composition in new-onset untreated RA (NORA) patients shaped by the increase of the abundance of *Prevotella copri* and the reduction of *Bacteroides* if compared with healthy subjects. By studying the pathogenicity of *P. copri* and its role in RA, these authors have discovered that this species is able to stimulate the T-helper 1 cells in the NORA patient group by the synthesis of a 27 kD protein, and then to negatively influence the outcome of the disease. This compound is indeed able to stimulate abundant rate of specific immunoglobulin A (IgA) and immunoglobulin G (IgG) antibodies [14]. However, this negative effect on RA seems strain-dependent. Interestingly, the study by Marietta et al. [15], performed in an experimental model of arthritis in mice, has indeed demonstrated that a new strain of the *Prevotella* genus, *Prevotella histicola*, isolated from the colon, counteracts the insurgence of arthritis and has a positive effect on RA. 

Regrettably, recent findings suggest that other species may also be involved as potential influencers in the clinical outcome of RA. The study of Chen et al. [12] correlated RA with the abundance of a gram positive called *Collinsella*, a genus of Actinobacteria present in the gut. Another studies conducted by Zhang et al. [16] found that the abundance of *Eggerthella lenta* and *Collinsella* correlated with RA outcomes, independently by diet or host genetic confounding factors. This suggests that RA originates at mucosal sites, which includes also the oral mucosal cavity. *Porphyromonas gingivalis*, a major pathogenic bacterium of periodontal diseases, is indeed recently considered to correlate with the development of RA with mechanisms similar to *P. copri* action in the mucosal gut [17,18]. 

Maeda and Takeda [19], supported by other additional authors [20], finally demonstrated that the mono-colonization of germ-free mice with *P. copri* was able to induce arthritis. The study was developed in a Th17 cell-dependent autoimmune arthritis, clinically resembling human RA, after injection of low doses of zymosan (a fungal component). Considering these experiments all together, it is therefore highly suggestive to think that dysbiosis dominated by *P. copri* in the gut contributes to RA development and its maintenance. 

The study of Pianta et al. [14] had already identified a specific HLA-DR-presented peptide ((Human Leukocyte Antigen – DR isotype of T cell epitope) in a 27 kDa *P. copri* protein, called Pc-p27, in the synovial environment [14]. This peptide was able to increase IL-17 production as well as the IgG and IgA anti-citrullinated antibody responses, as similarly occurs in RA. The study concluded that *P. copri* may then contribute to the development of RA. 

Thus, dysbiosis and particular bacterial clusters as *P. copri* first, but also *Collinsella*, *Erghetella*, the oral *P. gingivalis*, and others, such as the segmented filamentous bacteria (SGB) [21], or a particular strain of *Lactobacillus bifidus* [22] can play an important role in the pathogenesis of RA.

Further studies have evidenced that this type of dysbiosis is correlated not only to the increase of the above-mentioned microorganisms’ abundance, but also to the decrease of *Bacteroides*, *Veillonella*, *Eubacterium*, or *Haemophilus* genres in the gut [9,13,16,20]. This is a clear demonstration that the unbalance of some combined and specific microbial clusters may be responsible for the pathogenesis of RA. 

Thus, the presence of predisposing genetic and environmental host factors combined with a particular altered profile of the gut microbiota (also oral) may lead to an increased risk of RA.

A very recent study reveals that gut microbiome dysbiosis can be restored in a eubiotic status after administration of the so called “disease-modifying anti-rheumatic drugs” (DMART) [23]. This study indirectly confirms the important role of microbiota balance in the influence of RA disease and that some anti-rheumatic drugs can have a “probiotic” effect by inducing host microbiota modulations to generate a gut eubiosis. This and other studies have opened the way for the possibility of using real bacterial probiotics (living microorganisms that upon consumption in adequate amounts can improve the health of the intestinal microbial flora) as potential gut restoring tools to rebalance the alteration of the microbiota [24,25]. 

Although the studies on RA and gut microbiota need to be further corroborated by new and strong evidence, the finding that a *P. copri*-dominated microbiota combined with genetic and other external RA-influencing factors may represent a further risk factor for the development of the disease is highly suggestive. Thus, looking at *P. copri* as fecal marker along with a specific metagenomic gut dysbiotic profile in the RA patents could be a good intriguing field for better understanding the disease outcome. 

More intensive human studies and in-depth in vivo experiments will surely be needed to investigate whether microbiota, or other than *P. copri* bacteria and specific microbial clusters can elicit severe arthritis. 

In conclusion, future perspectives are mandatory to identify the precise biomolecular links between *P. copri* gut dysbiosis (or *P. gingivalis* oral dysbiosis) and the onset and maintenance of human RA. The final aim will be to develop novel therapeutic/preventive approaches as well as to study additional biological markers in RA patients by harnessing the microbiota of the body.
